# Loss of Control Increases Belief in Precognition and Belief in Precognition Increases Control

**DOI:** 10.1371/journal.pone.0071327

**Published:** 2013-08-07

**Authors:** Katharine H. Greenaway, Winnifred R. Louis, Matthew J. Hornsey

**Affiliations:** The University of Queensland, School of Psychology, Brisbane, Queensland, Australia; George Mason University/Krasnow Institute for Advanced Study, United States of America

## Abstract

Every year thousands of dollars are spent on psychics who claim to “know” the future. The present research questions why, despite no evidence that humans are able to psychically predict the future, do people persist in holding irrational beliefs about precognition? We argue that believing the future is predictable increases one’s own perceived ability to exert control over future events. As a result, belief in precognition should be particularly strong when people most desire control–that is, when they lack it. In Experiment 1 (*N* = 87), people who were experimentally induced to feel low in control reported greater belief in precognition than people who felt high in control. Experiment 2 (*N* = 53) investigated whether belief in precognition increases perceived control. Consistent with this notion, providing scientific evidence that precognition is possible increased feelings of control relative to providing scientific evidence that precognition was not possible. Experiment 3 (*N* = 132) revealed that when control is low, believing in precognition helps people to feel in control once more. Prediction therefore acts as a compensatory mechanism in times of low control. The present research provides new insights into the psychological functions of seemingly irrational beliefs, like belief in psychic abilities.

## Introduction

The human mind is predisposed toward prediction. We are constantly driven to look forward, envisage the future, and infer what will happen [1]. These cognitive mechanisms serve important functions in enabling survival and reproductive advantage, and also act to reduce psychological uncertainty about the future. Our natural orientation towards prediction can sometimes manifest in extreme ways, with some people going so far as to postulate that humans may have developed an ability to predict the future. The multi-million dollar industry of psychic readings, clairvoyance, and astrology testifies to people’s fascination with this idea. One in four Americans believes in precognition [2]. Even the attention of the scientific community has been captured by recent claims that precognition exists [3], [4]. It seems strange considering humans are more scientifically and intellectually advanced than ever before, that irrational beliefs about precognition can persist and be maintained. The present research seeks to explain this phenomenon.

### Psychological Pull of Paranormal Beliefs

It is clear that people are drawn to the idea that it is possible to psychically predict the future, that is, access information about what will happen before it has happened. What is less well understood is *why* people hold these beliefs, and what function these beliefs serve. Although precognitive abilities would confer decided adaptive advantages from an evolutionary perspective, current scientific knowledge indicates that humans do not and probably cannot predict the future through psychic means [5], [6]. From a purely rational perspective, it is unclear what benefit people might gain from believing in something that does not exist. From a psychological perspective, it makes sense that beliefs that help people attain a desired end state such as feeling happy, safe, and secure will be differentially endorsed.

Even the most extreme beliefs may be beneficial when they help people deal with stress or threat. For example, superstitious beliefs and behaviors such as wearing a “lucky charm” have been found to enhance performance [7], to protect against negative outcomes under stress [8], [9], and to be positively correlated with feelings of control [10], [11]. Sports players use superstitious strategies to attempt to bring about a desired outcome in a game [12], [13]. Times of economic stress reliably coincide with an increase in the number of articles on astrology and other psychic phenomena [14]. Many Western societies may be experiencing another such peak in the wake of the Global Financial Crisis and the events of September 11, 2001 [15]–[17].

Stressful or challenging experiences therefore often seem to go hand-in-hand with interest in paranormal phenomena. Some researchers even argue that paranormal beliefs, and belief in precognition in particular, may develop as part of a specialized psychological coping mechanism in the service of managing feelings of threat [18], [19], [20]. Specifically, such beliefs are thought to aid people in threatening circumstances by conferring a sense of control.

If it is possible to predict what the future holds, then one can exert control. That is, by knowing what will happen, people can act in a way to bring about positive outcomes and avoid negative outcomes. For instance, one could earn money by knowing the outcome of a particular sporting match, or remain safe by choosing to avoid the site of a future accident. Having insight into what will happen in the future would therefore allow people to control their outcomes in a way that would guarantee personal success and survival. Accordingly, we argue that believing the future is predictable, even through psychic means, should increase people’s perceived ability for control.

A wealth of correlational evidence exists to show that belief in precognition is positively associated with perceived control [19]–[21], [23]. It is clear, therefore, that belief in precognition and control are reciprocally intertwined. Yet, the existence of a correlation does not provide information about whether belief in precognition provides people with a sense of control, or whether challenges to control lead to the endorsement of precognitive beliefs, or whether both processes operate. The present research tested these relationships experimentally.

### Compensating for Compromised Control

Humans are motivated to feel in control of their environments and outcomes [24], [25]. The impulse is unsurprising, given that people who experience high perceived control tend to live happier, healthier, and more productive lives than people who do not [25]–[29]. As a result of this drive, people act in order to regain perceived control when deprived of it [24], [30], [31]. Accordingly, humans have developed a variety of psychological strategies to combat feelings of uncontrollability.

When control is deprived, people may attempt to regain it through primary or secondary means. A primary method would be to change the situation that engendered feelings of uncontrollability [24], [31]. Alternatively, people may engage in secondary–or compensatory–strategies to increase perceived control [32]–[40]. Secondary control strategies are often used when people lack actual control over their circumstances. They involve changing one’s desires to fit with the current circumstances, rather than changing the circumstances to fit with one’s desires (i.e., primary control). To this end, individuals adjust their own attitudes, beliefs, and desires to help themselves feel more in control of an uncontrollable situation.

Rothbaum and colleagues [24] published a comprehensive review of secondary strategies that people use to cope with situations of uncontrollability. One secondary response that they outlined is a strategy termed *predictive control*, in which people strive to predict future events in order to be able to better exert control. Some scholars have even defined desire for control as the motive to “render the world predictable” ([41], p.551). If a predictive control strategy is utilized, then beliefs that are associated with predictability should be preferentially favored when people find themselves in situations of low control.

### Precognition as Predictive Control

Precognitive abilities would allow people to predict the future, thus belief in these abilities should be differentially endorsed when people most desire prediction–that is, in situations of low control. We posit therefore that belief in precognition is a predictive control strategy that people can turn to when feeling low in control. As a result, we hypothesize that loss of control will cause an increase in belief in precognition. Certainly loss of control has been found to increase other types of paranormal beliefs–like superstition–which also include an element of being able to predict, or at least guide, the future [11]. In the case of precognition, people have a direct and exact channel to knowing the future through psychic means. These types of beliefs should therefore be particularly attractive as predictive control strategies in so far as they give people the illusion of being able to predict (and therefore control) the future.

The present research aimed first to determine whether loss of control increases belief in precognition. Our second aim was to determine whether these beliefs do indeed serve as a predictive control strategy, by testing whether belief in precognition increases perceived control. In combining these research questions we will be theoretically advancing the control literature. To date, control researchers have focused on cataloguing the range of strategies people engage in when low in control [11], [32], [35], [39], [40]. Implicit in this literature is the assumption that these compensatory control strategies act to restore perceived control when it has been lost. Research suggests that control strategies do serve this function: They are associated with feelings of control [42], reduce anxious arousal when control has been depleted [37] and can help to meet a need for order and structure [38]. Although this evidence is suggestive, it still requires concrete evidence that engaging in control strategies can act to increase perceived control after a direct loss of control.

The present research aimed to show that the psychological strategies people engage in when low in control can and do serve to increase perceived control. This work therefore provides a new theoretical lens through which to view belief in precognition as more than just an irrational indulgence. It reveals that such beliefs are not necessarily an irrational response to loss of control, but serve a psychological purpose of boosting perceived control in times of uncontrollability.

## Experiment 1

Experiment 1 investigated the effect of loss of control on belief in precognition. Given the existence of a positive correlation between perceived control and belief in precognition, there are two possible directions that this effect might take. First, if control and precognition have a straightforward one-to-one relationship, then depriving people of control could reduce their endorsement of precognitive abilities. However, another relationship is possible, that depriving people of control may *increase* belief in precognition. This is because the existence of a positive association does not preclude the possibility that reducing one variable will trigger an increase in the other variable to take its place [36]. Such a hydraulic effect would be consistent with theorizing by Irwin and colleagues [19], [20] that paranormal beliefs like precognition develop as part of a coping mechanism to help people manage threat. It would also parallel findings by Kay and colleagues of hydraulic relationships between cognitions that provide order and stability following a loss of control [36]. To test whether loss of control increases or decreases belief in precognition, Experiment 1 measured these beliefs after people were exposed to a manipulation designed to prime feelings of high control or low control.

### Method

#### Ethics Statement

The School of Psychology Student Research Ethics Review at the University of Queensland, Australia approved the procedures for the experiment. It is the policy of this Review Committee to obtain verbal rather than written consent and as such, participants provided verbal informed consent, recorded by the experimenter on a written log sheet.

#### Participants and Procedure

Eighty-five first-year psychology students participated in the experiment in exchange for partial course credit (63 female; *M_age_ = *18.61, *SD = *2.75). Control (high vs. low) was manipulated via a priming task in which participants recalled and wrote about a time they felt in control or a time that they felt they had no control [11], [35]. This experiment was part of a larger project that also manipulated financial threat (high vs. low) in the context of the Global Financial Crisis. There were no significant main (*p* = .684) or interactive effects (*p* = .165) of the threat manipulation on belief in precognition.

Belief in precognition was measured using four items from the Revised Paranormal Belief Scale ([46], “Some people have an unexplained ability to predict the future”; “Astrology is a way to accurately predict the future”; “The horoscope accurately tells a person’s future”; and “Some psychics can accurately predict the future”; α = .74) measured on a scale ranging from 1 (*strongly disagree*) to 7 (*strongly agree*).

### Results and Discussion

The relevant data and syntax reported in this paper are available from the authors by request. As predicted, there was a significant effect of the control manipulation, *F*(1,83) = 4.93, *p = *.029, η_p_^2^ = .056. Participants who recalled a time of low control reported greater belief in precognition (*M* = 2.75, *SD* = 1.27) than participants who recalled a time of high control (*M* = 2.17, *SD* = 1.12). This finding provides experimental evidence that people who feel low in control report believing in precognition more so than people who feel high in control. It is in these uncontrollable contexts that people most crave the comfort of knowing that the future is predictable.

The results echo findings by Whitson and Galinksy [11] that threats to control increase superstition, beliefs about events that cause bad luck. It is not surprising that precognitive beliefs would show a similar pattern to superstitious beliefs. First, correlational research has shown belief in superstitions and belief in precognition to be highly correlated [44]. Most superstitions share a common underlying theme of rendering people’s lives more predictable (i.e., both are predictive control strategies [24]). Nevertheless, the two beliefs are not isomorphic. Experiment 1 demonstrated an effect of control deprivation on precognition specifically.

In the following two experiments, our research goes beyond previous experimental work that demonstrates that loss of control heightens paranormal beliefs to test why this effect occurs. Specifically, in the following two experiments we tested our hypothesis that belief in precognition actually boosts perceived control and for this reason is endorsed to a greater degree when people feel as though they lack control.

## Experiment 2

Researchers have argued that people use secondary strategies when control has been threatened because these strategies act to restore or otherwise compensate for loss of control [32]–[37]. More specifically, researchers have hypothesized that paranormal beliefs like precognition provide people with an enhanced sense of control [5], [19]–[22]. It seems plausible therefore that people are drawn to a belief in precognition because it provides them with a heightened sense of control.

To test whether belief in precognition increases perceived control, Experiment 2 included a manipulation designed to increase belief in precognition. We exploited a recent debate in the psychology literature to test this research question. Recently, Daryl Bem [3] published an article in the premier social psychology journal, the *Journal of Personality and Social Psychology*, reporting scientific evidence for the existence of precognition. The paper inspired much attention and controversy, including a rebuttal article by Wagenmakers and colleagues [4] published in the same issue of *JPSP*. In Experiment 1, half of the participants read the abstract of Bem’s article showing experimental evidence for precognition. The other half read the abstract by Wagenmakers and colleagues debunking the notion that precognition exists. We hypothesized that people who read Bem’s claim that precognition exists would report greater perceived control than people who read the alternative claim that it does not exist.

### Method

#### Ethics Statement

The School of Psychology Student Research Ethics Review at the University of Queensland, Australia approved the procedures for the experiment. It is the policy of this Review Committee to obtain verbal rather than written consent and as such, participants provided verbal informed consent, recorded by the experimenter on a written log sheet.

#### Participants and Procedure

Fifty-three participants (33 female; *M_age_ = *18.96, *SD* = 3.01) were approached on the campus of a large Australian university and asked to participate in the experiment in exchange for a chocolate bar. Participants in the *precognition condition* read a paragraph stating that researchers had found evidence for the existence of precognition. Featured below the paragraph was the abstract of Bem’s [3] article on this topic. Participants in the *no precognition condition* read that researchers had debunked the notion that precognition exists. Featured below the paragraph was the abstract of the rebuttal article by Wagenmakers and colleagues [4].

Following the precognition manipulation, perceived control was measured using three items (“I am in control of my own life”; “I am able to live my life how I wish”, “My life is determined by my own actions”, α = .76) on a scale ranging from 1 (*strongly disagree*) to 7 (*strongly agree*).

### Results and Discussion

As predicted, there was a significant effect of the precognition manipulation on perceived control, *F*(1,51) = 4.19, *p = *.046, η_p_^2^ = .076. Participants who read that precognition exists reported higher perceived control (*M* = 5.37, *SD* = 0.61) than participants who read that precognition does not exist (*M* = 4.75, *SD* = 1.41).

Experiment 2 demonstrated experimentally that informing people that precognition exists increases perceived control relative to informing them that precognition does not exist. This effect augments previous correlational findings of a positive association between belief in precognition and perceived control [21], [23]. Moreover, it helps to clarify why people are more likely to adopt paranormal beliefs of this sort in times of uncontrollability–because these beliefs act to increase perceived control. The finding supports theorizing that such beliefs are protective and help people to cope with loss of control [18]–[20]. To provide concrete evidence for this theorizing, however, it is necessary to test whether feelings of control are protected if people endorse a belief in precognition after experiencing a loss of control. The final experiment tested this full model that belief in precognition boosts perceived control particularly in times of uncontrollability.

## Experiment 3

Experiment 1 provided evidence that loss of control increases belief in precognition. Experiment 2 demonstrated that belief in precognition increases perceived control. Experiment 3 combined these observations and tested a full theoretical model of control restoration. The main argument we put forward is that when people lack control, believing in precognition helps them to feel in control once more.

In generating this hypothesis, we drew on theorizing and research in the control literature. A weight of empirical evidence indicates that people seek to restore perceived control when it has been deprived [24], [30], [31], [47], [48]. More recent research has documented a range of strategies that people use to compensate for a lack of control [11], [32], [35], [39], [40]. We propose that heightened belief in precognition is another such compensatory strategy. Implicit in the control literature is the idea that people engage in these compensatory strategies because they act to increase feelings of control. We aimed to directly test whether these types of control strategies do indeed boost perceived control.

In order to demonstrate this full model, Experiment 3 manipulated both control and belief in precognition as in the previous experiments. We then measured subsequent ratings of perceived control. We hypothesized that belief in precognition would increase perceived control when participants were induced to feel low in control (but not when they were induced to feel high in control). The present research therefore directly tests whether compensatory control strategies act to boost perceived control. In addition to this theoretical advancement, Experiment 3 made a methodological improvement on Experiment 2 by including a baseline condition–a condition in which there was no mention of precognition–to compare against the precognition and no precognition conditions.

### Method

#### Ethics Statement

The School of Psychology Student Research Ethics Review at the University of Queensland, Australia approved the procedures for the experiment. It is the policy of this Review Committee to obtain verbal rather than written consent and as such, participants provided verbal informed consent, recorded by the experimenter on a written log sheet.

#### Participants and Design

One hundred and thirty-two students (83 female; *M_age_* = 20.28, *SD* = 4.34) completed the experiment in exchange for course credit. The experiment employed a 2 (induced low control vs. induced high control)×3 (precognition vs. no precognition vs. baseline) design with perceived control as the dependent variable.

#### Manipulations and Measures

Control was manipulated using the priming task from Experiment 1 in which participants recalled and wrote about a time they experienced *control* or *no control*. The precognition manipulation was similar to that in Experiment 2. Participants in the *precognition* condition read the abstract by Bem [3] with an explanatory paragraph titled “Precognition exists, psychologists find”. Participants in the *no precognition* condition read the abstract by Wagenmakers and colleagues [4] with an explanatory paragraph titled “Precognition does not exist, psychologists find”. We also added a *baseline* condition in which participants read the abstract of an article from the same issue of *JPSP* by Mahajan and colleagues [49]. The paper revealed that rhesus macaques can discriminate between members of their own and other social groups and was preceded by an explanatory paragraph titled “Psychologists find social discrimination in monkeys”.

Following the manipulations, perceived control was measured using five items (“I am in control of my own life”; “I am free to live my life how I wish”; “My life is determined exclusively by my own actions”; “I enjoy making my own decisions”; and “I enjoy having control over my own destiny”; α = .73) on a scale ranging from 1 (*strongly disagree*) to 7 (*strongly agree*). Analyses using only the first three items (replicating the perceive control scale from Experiment 2) yields a significant interaction, *F*(2,126) = 3.94, *p = *.022, η_p_^2^ = .059, which follows the same pattern of results as those reported in the results section below.

### Results and Discussion

There was no significant main effect of the control manipulation, *F*(1,126) = 0.09, *p = *.772, η_p_^2^ = .001, or the precognition manipulation, *F*(2,126) = 0.60, *p = *.549, η_p_^2^ = .009. However, as expected, there was a significant interaction between the control and precognition manipulations, *F*(2,126) = 3.41, *p = *.036, η_p_^2^ = .051.

Simple effects revealed that the effect of the precognition manipulation was not significant in the high control condition, *F*(2,126) = 0.76, *p = *.472, η_p_^2^ = .012. In line with predictions, the effect of the precognition manipulation was significant in the low control condition, *F*(2,126) = 3.25, *p = *.042, η_p_^2^ = .049. Simple comparisons revealed that within the low control condition, people in the precognition condition reported greater levels of perceived control (*M* = 5.76, *SD* = 0.66) than people in the no precognition condition (*M* = 5.19, *SD* = 0.69), *p* = .026, and the baseline condition (*M* = 5.21, *SD* = 1.09), *p* = .030; see Table 1.

**Table 1:** Means and simple comparisons of the effect of precognition condition within the low and high control conditions.

	No precognition	Baseline	Precognition
Low control	5.19_a_	5.21_a_	5.76_b_
High control	5.36_a_	5.49_a_	5.18_a_

Means with different subscripts indicate significant comparisons.

Inspection of the alternative set of simple effects revealed that the effect of control was non-significant in the no precognition condition, *F*(1,126) = 046, *p = *.501, η_p_^2^ = .004, and the baseline condition, *F*(1,126) = 1.28, *p = *.261, η_p_^2^ = .010, but was significant in the precognition condition, *F*(1,126) = 5.15, *p = *.025, η_p_^2^ = .039. Within the precognition condition, participants in the low control condition reported greater perceived control (*M* = 5.76, *SD* = 0.66), than participants in the high control condition (*M* = 5.18, *SD* = 0.87).

As expected, participants who were told that precognition exists reported feeling more in control compared to baseline participants or participants who were told that precognition does not exist. However, this effect emerged only when people were induced to feel low in control. When control was affirmed by having people recall a time that they felt capable and in charge, there was no effect of belief in precognition on perceived control. Under such conditions, people have no need for belief systems that will boost feelings of controllability, because they already feel capable of exerting control. It is when control is deprived that people cling to the belief that the future is predictable, and under such conditions holding this belief is palliative and functional in that it increases perceptions of control.

## General Discussion

The present research demonstrated that loss of control increases belief in precognition and belief in precognition increases perceived control. Our first step was to demonstrate that when people lack control they report greater belief in precognition. In Experiment 1, people who were experimentally induced to feel low in control were more likely to believe in precognitive abilities. These findings add belief in precognition to the long list of compensatory control strategies that people use under conditions of uncontrollability [11], [32], [35], [39], [40].

Confirming long-standing observations of a correlational relationship between control and precognition [19], [23], [43], [45], Experiment 2 revealed that perceived control was higher when people were told that precognition exists relative to when they were told that precognition does not exist. Experiment 3 provided evidence for a full theoretical chain of control restoration. When control was deprived, belief in precognition increased the perception that one is in control of their life.

This finding makes an important addition to the compensatory control literature. There is a widespread assumption in the literature, supported indirectly, that many of the secondary strategies people engage in after a loss of control are in fact designed to increase perceived control. We made a direct test of this hypothesis, and demonstrated concretely that these strategies can succeed in increasing perceived control.

This work provides evidence that belief in precognition is a novel type of predictive control strategy [24]. Our findings demonstrate that people are drawn to prediction when they lack control, even when that prediction involves acknowledging the existence of paranormal abilities like precognition. This is because if the future is predictable, it can be controlled; a belief that is particularly attractive to people when they feel deprived of control.

The data in the present paper show that, on average, people tend to react to situational loss of control with heightened belief in precognition. In such contexts, precognitive belief is a reactive attempt to boost perceived control when it has been threatened. The fact that a positive correlation exists in the general population suggests the hypothesis that the boost in control may be short-lived. There may even be a distal cost to embracing a belief in precognition specifically as a defence against control deprivation. If this belief is subsequently disconfirmed, the threat to control may be even more intense. Longitudinal research is needed to identify how long the sensation of boosted control derived from reactive paranormal beliefs might last, and whether there is a rebound of vulnerability to threatened control in the longer term.

### Conclusions

The present research has shown that beliefs about psychic predictability can provide the psychological system with a compensatory boost in perceived control. We found that people were drawn to predictability when they experienced loss of control–even to the extent of endorsing seemingly irrational beliefs about precognition. We propose, therefore, that these kinds of beliefs are not an unreasonable response to control deprivation. Indeed, to the extent that belief in precognition increases perceived control, people could be described as becoming *functionally* irrational by holding this or related beliefs when control is threatened.

On a practical note, our findings help to explain why interest in the predictive arts is highest in times of threat and uncertainty [12]–[17]. It is at these moments that individuals most feel the need to control the course of their lives. Belief in precognition meets this need by enabling people to feel that the future is predictable, and can therefore be controlled. Regardless of whether precognitive abilities actually exist, therefore, belief in their existence serves an important psychological function of boosting perceived control in times of uncertainty.
